# New Appliances and Things Medical

**Published:** 1895-03-23

**Authors:** 


### NEW APPLIANCES AND THINGS MEDICAL.

[We shall be glad to receive, at our Office, 428, Strand, London, W.C., from the manufacturers, specimens of all new preparations and appliances which may be brought out from time to time.]

### RICH OAT CAKES.

(MacFarlane, Lang, and Co, Victoria Biscuit Factory, Glasgow.)

We have received a sample of the above for notice. To the palate nothing could be nicer, especially after the cakes have been warmed through in the oven as directed on the cover of the box. Examined chemically the result is equally good, absence of fibre, husk, or other extraneous material so common in ordinary oat preparations and also of deleterious additions of any kind show the skill and care with which these cakes are manufactured. We cordially welcome them as a new and very pleasant addition to the biscuit tribe.

### COCKATOO SOAP.

(F. L. Bartelt, Keynsham, near Bristol.)

This soap does not seem to differ from many other kinds already in the market, notwithstanding the fact that it is described as "the newest and most wonderful invention." The soap is guaranteed free from resin and to be made only from the purest fats. It does not, however, float on water as stated on the tablet, and this is probably due to the fact that in remelting enough air bubbles have not been agitated into the mass. We are of the opinion that all soaps which are advertised as floating on water should be bought by weight, as their lightness is invariably due to the presence of air bubbles, which give the soap a spongy texture and increases its apparent volume. We must also take exception to the statement of the manufacturer that "Cockatoo" Soap is absolutely disinfecting and absolutely antiseptic. There is no reason why the soap itself should not be aseptic, but it is obvious that if it were disinfecting it would also be antiseptic. We have not, however, discovered anything in the soap which can be called a germicide. There was no free alkali in the sample sent us for examination, but this seems to be due to the fact that the soap had not been properly boiled, as there was upwards of eight per cent. of unsaponified fats in the sample. The amount of water present was 26·67 per cent., an amount which, although high, cannot be regarded as excessive until there is some authoritative opinion or legislative action on the quantity of water which may normally be allowed in a well-manufactured soap.

